# Diagnostic and predictive value of speckle tracking echocardiography in cardiac sarcoidosis

**DOI:** 10.1186/s12872-019-01323-0

**Published:** 2020-01-20

**Authors:** Cristina Di Stefano, Giulia Bruno, Maria C. Arciniegas Calle, Gayatri A. Acharya, Lynn M. Fussner, Patompong Ungprasert, Leslie T. Cooper, Lori A. Blauwet, Jay H. Ryu, Patricia A. Pellikka, Eva M. Carmona Porquera, Hector R. Villarraga

**Affiliations:** 1 Department of Cardiovascular Medicine, Mayo Clinic, 200 First St SW, Rochester, MN 55905 USA; 2 Division of Pulmonary and Critical Care Medicine, Mayo Clinic, Rochester, MN USA; 3 Division of Rheumatology, Mayo Clinic, Rochester, MN USA; 4 Department of Cardiovascular Diseases, Mayo Clinic, Jacksonville, FL USA

**Keywords:** Cardiac sarcoidosis, Echocardiography, Speckle tracking, Strain

## Abstract

**Background:**

Sarcoidosis is a systemic granulomatous disease that may affect the myocardium. This study evaluated the diagnostic and prognostic value of 2-dimensional speckle tracking echocardiography in cardiac sarcoidosis (CS).

**Methods:**

Eighty-three patients with extracardiac, biopsy-proven sarcoidosis and definite/probable diagnosis of cardiac involvement diagnosed from January 2005 through December 2016 were included. Strain parameters in early stages of CS, in a subgroup of 23 CS patients with left ventricular ejection fraction (LVEF) within normal limits (LVEF> 52% for men: > 54% for women, mean value: 57.3% ± 3.8%) and no wall motion abnormalities was compared with 97 controls (1:4) without cardiac disease. LV and right ventricular (RV) global longitudinal (GLS), circumferential (GCS), and radial (GRS) strain and strain rate (SR) analyses were performed with TomTec software and correlated with cardiac outcomes (including heart failure and arrhythmias). This study was approved by the Mayo Clinic Institutional Review Board, and all patients gave informed written consent to participate.

**Results:**

Mean age of CS patients was 53.6 ± 10.8 years, and 34.9% were women. Mean LVEF was 43.2% ± 12.4%; LV GLS, − 12.4% ± 3.7%; LV GCS, − 17.1% ± 6.5%; LV GRS, 29.3% ± 12.8%; and RV wall GLS, 14.6% ± 6.3%. In the 23 patients with early stage CS with normal LVEF and RV systolic function, strain parameters were significantly reduced when compared with controls (respectively: LV GLS, − 15.9% ± 2.5% vs − 18.2% ± 2.7% [*P* = .001]; RV GLS, − 16.9% ± 4.5% vs − 24.1% ± 4.0% [*P* < .001]). A LV GLS value of − 16.3% provided 82.2% sensitivity and 81.2% specificity for the diagnosis of CS (AUC 0.91), while a RV value of − 19.9% provided 88.1% sensitivity and 86.7% specificity (AUC 0.93). Hospital admission and heart failure significantly correlated to impaired LV GLS (> − 14%).

**Conclusion:**

Reduced strain values in the LV GLS and RV GLS can be used in the diagnostic algorithm in patients with suspicion of cardiac sarcoidosis. These values also correlate with adverse cardiovascular events.

## Background

Sarcoidosis is a systemic granulomatous disease that can affect the myocardium. Myocardial granulomas are detected post-mortem in 12 to 25% of patients with extracardiac sarcoidosis [1, 2]. These granulomas lead to subclinical myocardial inflammation, edema, fibrosis, and scars, which in turn result in ventricular remodeling and systolic dysfunction [3]. The prognosis of cardiac sarcoidosis (CS) is poor; a 5-year mortality rate of 25 to 40% in the advanced stages of CS is attributable to arrhythmias, restrictive cardiomyopathy, and sudden cardiac death [4, 5]. Myocardial involvement is clinically silent and may be underdiagnosed [6–8]. The gold standard for diagnosing CS is endomyocardial biopsy. However, its sensitivity is low because of the patchy distribution of granulomas in both the left and right ventricles [9]. Conventional echocardiography may identify typical myocardial alterations due to sarcoidosis, such as wall aneurysms, wall motion abnormalities, and thinning of the basal septum, but it may underestimate regional myocardial dysfunction since in the early phases of the disease it can appear normal [10]. Furthermore, cardiac magnetic resonance (CMR) and fluorodeoxyglucose–positron emission tomography (FDG–PET) imaging may be useful to detect edema, active inflammation or scars in myocardial walls [11] but they are not always available.

Two-dimensional–speckle tracking echocardiography (2D–STE) is a promising method that is sensitive for early detection of cardiac dysfunction in systemic diseases, including CS.

The aim of this study was to describe 2D–STE and conventional echocardiographic parameters of left ventricular (LV) and right ventricular (RV) systolic and diastolic function in patients affected by CS, compare the results with age- and sex-matched patients without CS, and evaluate the prognostic value of 2D–STE parameters on cardiac events.

## Methods

We retrospectively identified 122 patients with systemic sarcoidosis that were evaluated at our Department of Cardiovascular Medicine and had a transthoracic echocardiogram performed for suspected CS from January 1, 2005 through December 31, 2016. All CS patients were referred to echocardiographic evaluation due to symptoms (included cardiac arrhythmias, chest pain, fatigue and dyspnea), EKG alterations or for a cardiologic consult. The diagnosis of definite or probable CS was made by myocardial biopsy or in accordance with clinical criteria of the Heart Rhythm Society (HRS) consensus statement [12] in 83 (68%) of patients; which represents our study group. Findings from CMR were considered diagnostic when patchy myocardial areas of delayed gadolinium enhancement suggested scars or chronic fibrosis or when there was increased signal intensity on T2-weighted sequences with a non-coronary distribution, suggestive of active inflammation [13, 14]. Diagnostic FDG–PET imaging included increased, focal myocardial FDG uptake without altered perfusion findings [13]. Except for 2 patients with isolated CS, all patients had histologic findings of extracardiac, non-caseating granulomas and clinic-radiologic features consistent with sarcoidosis. Diagnosis was made after exclusion of infectious diseases, cardiomyopathies or adverse effects from medical treatments. Arrhythmias or alterations of atrioventricular or intraventricular conduction were assessed with a basal 12-lead electrocardiogram (ECG).

The control group consisted of 97 corrective patients with normal echocardiographic findings and without comorbidities identified during the same time frame as the CS cases. The Mayo Clinic Institutional Review Board approved the study and waived written informed consent for those who provided research authorization.

### Echocardiographic evaluation

Standard 2D transthoracic echocardiographic images were acquired with a Vivid 7 ultrasound system (GE Healthcare) or iE33 Ultrasound machine (Phillips Medical Systems), using an S5 transducer by an experienced cardiac sonographer. Conventional parameters were assessed according to current guidelines. Thinning of the basal interventricular septum was defined as a diameter ≤ 4 mm at 10 mm from the aortic annulus [15]. LV volumes and LV ejection fraction were assessed by the Simpson biplane technique from apical 2- and 4-chambers views, indexed for body surface area. Wall motion abnormalities were defined as hypokinesis or akinesis of the ventricular wall. LV diastolic function was evaluated per the current recommendations of the American Society of Echocardiography [16]. LV filling pressures were estimated from the ratio between E peak velocity and the average of septal and lateral e′ velocities of the mitral annulus; high LV filling pressures were defined as E/e′ > 14. RV systolic function was evaluated by the tricuspid annular longitudinal systolic velocity on TDI (S′) and fractional area change. Abnormal RV systolic function was defined as a fractional area change < 35% and/or S′ peak velocity < 0.09 m/sec. RV dilatation was defined as end diastolic RV diameter > 41 mm [17]. Right ventricular systolic pressure was noninvasively estimated by the maximum velocity of tricuspid regurgitation detected with continuous-wave Doppler. Plus the right atrial pressure estimated from the diameter and collapses index of the inferior vena cava.

### LV deformation parameters assessed with 2D–STE

2D–STE was performed according to the current recommendations [18]. For all patients, the region of interest analyzed was adjusted to cover at least 90% of the myocardial wall thickness. Image-Arena, v4.6, software (TomTec Imaging System, 2D-Cardiac Performance Analysis module) was used to perform offline 2D–STE of the left and right ventricles. Frame rates were optimized between 40 and 90 frames/second. Standard 2D images of the LV in the parasternal short-axis view at the midpapillary level and in the apical 4-, 2-, and 3-chamber views, and RV-focused 4-chamber views were acquired and stored for offline measurements. Endocardial borders were traced in the end-systolic frame, creating a region of interest after manual adjustment of the epicardial borders. Global circumferential (GCS), radial (GRS), and longitudinal strain (GLS), and SR at peak systole (SRs) and at early diastole (SRe) were assessed. LV myocardial longitudinal strain, SRs, and SRe were measured with the average of 16 segment values (6 basal, 6 mid, and 4 apical segments) from the 3–2-4-chamber apical views, following the 16-segment model of the American Society of Echocardiography guidelines. LV circumferential and radial strain, SRs, and SRe were obtained from the average of 6 segments of the LV in short-axis view at the papillary level. RV strain was assessed by the average of both septal and lateral-wall segmental values (6 total segments) as well as free-wall strain from the RV-focused, 4-chamber views, including both endocardial and epicardial borders. Images with more than 2 segments of low-quality tracking were excluded from the final analysis.

### Outcomes

Adverse events occurring after CS diagnosis, including supraventricular and ventricular arrhythmias (ventricular fibrillation, ventricular tachycardia, atrial fibrillation, advanced atrioventricular block), heart failure, [19] ischemic stroke, new valve disease required percutaneous intervention, new-onset acute coronary syndrome, ICD or PM implantation, hospital admission for cardiac complications, need of LV assist device, cardiac transplantation and death, were collected from the electronic medical records.

### Statistical analysis

All data were analyzed with JMP software, version 10.0 (SAS Institute Inc). The Shapiro-Wilks test was used for testing the normality of the data. Quantitative variables were expressed as mean values and standard deviations. Qualitative variables were expressed as absolute values and percentages. Between-group comparisons among groups were performed with the Student *t* test or Mann-Whitney test for quantitative variables and with the χ^2^ test or Fisher exact test for qualitative variables, as appropriate. A post-hoc test (Bonferroni adjustment) or Tukey-Kramer was used to compare groups. Comparison of continuous and categorical variables between patients with sarcoidosis and matched control patients without sarcoidosis was performed with a paired *t* test and McNamara test. Receiver operating characteristic (ROC) curve analysis was carried out to assess the diagnostic value of a strain parameter for CS. Logistic analysis with the estimation of odd ratios (OR) was conducted to evaluate the contribution of strain parameters on cardiac events and outcomes. A *P* value <.05 was deemed statistically significant.

Intra-class correlation coefficients (ICCs) with 95% confidence intervals (Cis) have been used in our research laboratory to evaluate intra-observer and inter-observer variability.

## Results

Among the 83 patients who met the histologic and clinical criteria of CS according to the guidelines of the HRS consensus statement, [12] 25 patients had a definite CS diagnosis (granuloma detected at endomyocardial biopsy) and 58 a probable CS diagnosis (24 with perfusion defects and FDG uptake on FDG-PETand 34 patients with positive CMR findings, in which 16 had an inflammatory pattern with increased signal intensity T2 sequences and 18 had patchy delayed enhancement with non-coronary distribution). The main symptoms associated with the diagnosis were dyspnea (52%), palpitations (21%), and chest pain (11%). The mean (range) age of the cohort at diagnosis of CS was 53.6 ± 10.8 (25–75) years, 34.9% were women, 14.5% were African American, 67.5% had lung involvement, and 68.7% had lymph node involvement. A few patients had eye (9.6%), skin (12.0%), or central nervous system (5.0%) involvement. The mean age of the patients at the initial diagnosis of extracardiac sarcoidosis was 50.6 ± 11.3 years, and myocardial involvement was detected after a median (range) of 26.3 (0–1.291) months. On ECG, 67 patients had the following arrhythmias: type 2 s-degree atrioventricular (AV) block (3 patients, 3.6%), third-degree AV block (22 patients, 26.5%), right bundle branch block (14 patients, 16.9), left bundle branch block (2 patients, 2.4%), and ST-segment and T-wave alterations (26 patients, 31.3%). Acute coronary syndrome or other cardiomyopathies not related to sarcoidosis were ruled out in this cohort of patients. Other general characteristics of the CS patients and comorbidities are shown in Table 1.

**Table 1:** Characteristics of the Patient Cohort With Cardiac Sarcoidosis^a^

Characteristic	Value (*N* = 83)
Systolic blood pressure, mm Hg	113.6 ± 18.8
Diastolic blood pressure, mm Hg	70.9 ± 10.7
Heart rate, bpm	72.7 ± 15.6
BMI, kg/m^2^	29.4 ± 6.1
Hypertension	31 (37.3)
Diabetes mellitus	16 (19.3)
Dyslipidemia	25 (30.1)
Coronary artery disease^b^	5 (6.0)
Coronary catheterization	54 (65.1)
Prior immunosuppressive treatment	57 (68.7)
Systemic corticosteroids	57 (68.7)
MMF	10 (12.0)
Positive endomyocardial biopsy	25 (30.1)
VT/VF before CS diagnosis	32 (38.5)
ICD before CS diagnosis	32 (38.5)
Pacemaker before CS diagnosis	38 (45.8)

Abbreviations: *bpm* beats per minute; *BMI* body mass index; *CS* cardiac sarcoidosis; *ICD* implantable cardioverter defibrillator; *MMF* mycophenolate mofetil; *VT/VF* ventricular tachycardia/ventricular fibrillation

^a^ Values are mean ± SD or No. (%)

^b^ Four patients with a previous diagnosis of stable coronary artery disease had subsequent histologic diagnoses of CS, and 1 had findings on cardiac magnetic resonance of patchy, active inflammation of the left ventricle in an area of the ventricle unaffected by previous ischemia; all had a final coronary angiogram that showed no substantial coronary stenosis

### Echocardiographic parameters

All 83 patients underwent transthoracic echocardiographic evaluation. Mean LV mass indexed for body surface area was 111.9 ± 32.3 g/m^2^ in men and 104.2 ± 29.8 g/m^2^ in women; mean RWT was 0.38 ± 0.08 in men and 0.37 ± 0.08 in women. Thirty-one patients (37.3%) had eccentric hypertrophy, and 8 (9.6%) had concentric hypertrophy. Of the patients, 27% had normal LVEF, and 13.3% had severe LV systolic dysfunction (LVEF< 30%). Wall motion abnormalities were detected in two-thirds of patients, in particular in the basal (up to 22%) and mid-segments of the LV (up to 33%). Parameters of LV diastolic function were abnormal in most patients (grade 1: 39.3%, grade 2: 23.8%, grade 3: 7.1%). Of the patients, 22.9% had abnormal RV systolic function, and 36.1% had RV dilatation. Thirty-six (43%) patients had pulmonary hypertension: Of those, 23 had concurrent sarcoid pulmonary involvement; and 3 had severe tricuspid regurgitation. Other echocardiographic parameters are shown in Table 2.

**Table 2:** Echocardiographic Parameters in the Patients With Cardiac Sarcoidosis

Parameter	Value (*N* = 83)
Interventricular septum, mm	10.5 ± 2
LV diastolic volume indexed for BSA, mL/m^2^	79.4 ± 28.3
LV mass indexed for BSA, g/m^2^	111.9 ± 32.3
LV ejection fraction, %	43.2 ± 12.4
LA volume, mL/m^2^	32.6 ± 13.4
E/A	1.19 ± 0.63
E/e′	13.4 ± 10.1
Fractional area change, %	38.5 ± 12.5
Tricuspid annular S′, m/s	0.12 ± 0.03
RVSP, mm Hg	36.7 ± 13.1

Abbreviations: *BSA* body surface area; *E/A* ratio of early (E) to late (A) ventricular filling velocity; *E/e′* ratio between early mitral inflow velocity and mitral annular early diastolic velocity; *LA* left atrial; *LV* left ventricular; *RVSP* right ventricular systolic pressure; *S′* peak systolic annular velocity

Typical findings for advanced CS were found in 15 patients: 11 (13.3%) had interventricular septal thinning; and 4 (4.9%), wall aneurysms; all of these patients had severe LV and RV systolic dysfunction.

### Strain analysis

**Fig. 1:**
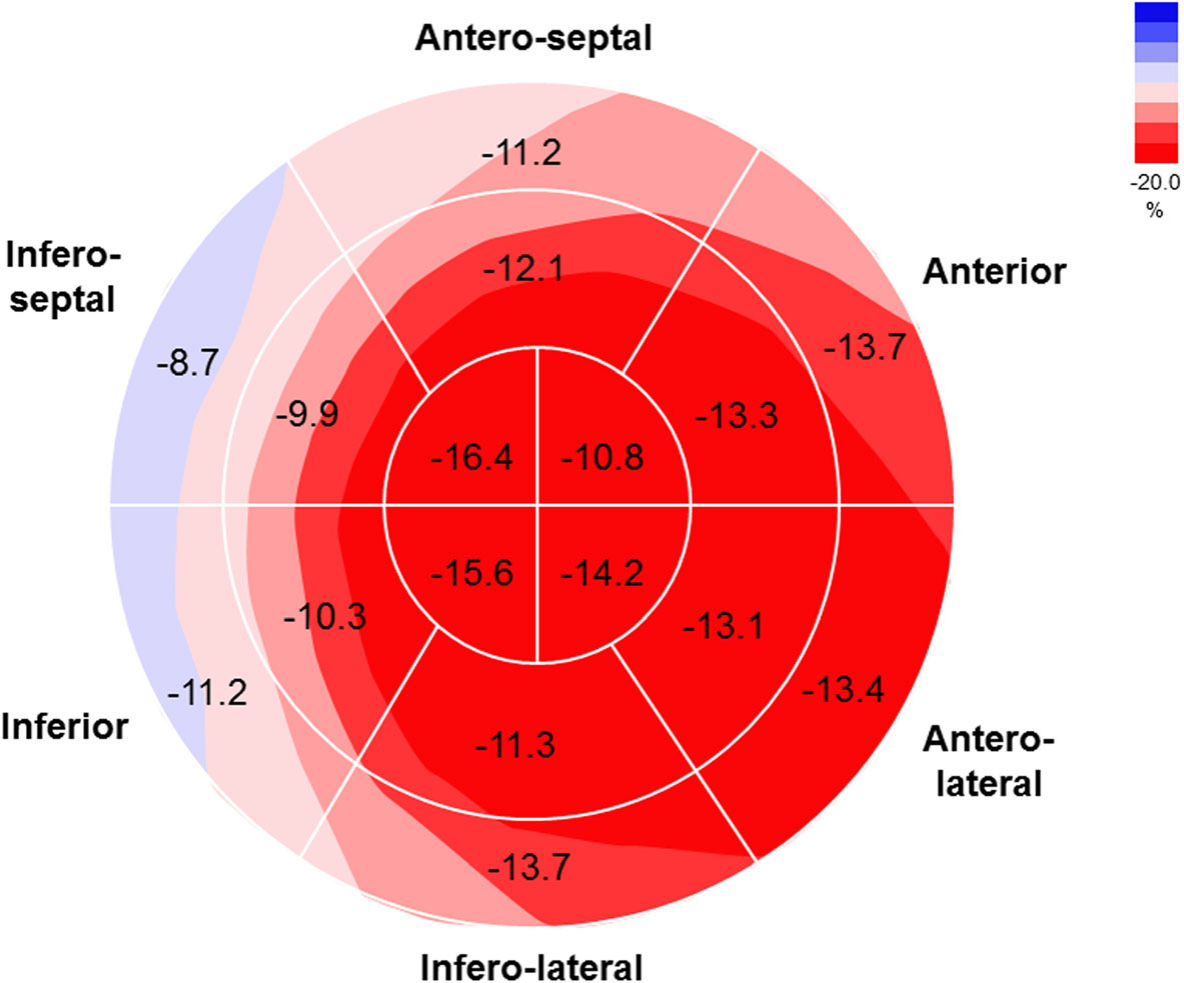
Bull’s eye display of mean left ventricular segmental longitudinal strain of 83 patients affected by cardiac sarcoidosis. All values are expressed in %

Mean LV GLS, GCS and GRS and RV GLS of CS patients are shown in Table 3. The percentage of reliability of 2D-STE analysis was 91% for longitudinal strain, 89% for circumferential and radial strain. Fig. 1 shows representative bulls eye display of LV segmental longitudinal strain; the lowest mean values were detected in the LV basal and mid interventricular inferoseptum as well as in the inferior wall. CS patients with a positive endomyocardial biopsy had more compromised LV GLS (− 10.5% ± 3.7), GCS (− 13.3% ± 6.7), global longitudinal systolic strain rate (GLSRs) (− 0.6 ± 0.2 s^− 1^), and global circumferential systolic strain rate (GCSRs) (− 0.8 ± 0.5 s^− 1^) as well as lower global longitudinal early diastolic strain rate (GLSRe) (0.6 ± 0.2 s^− 1^) and global circumferential early diastolic strain rate (GCSRe) (0.9 ± 1.3 s^− 1^) values; furthermore, RV and free wall RV GLS were also reduced more in this group of patients (− 11.5% ± 4.5 and − 13.0% ± 4.4, respectively). In addition, no differences in strain parameters were detected for patients who had never been treated compared with those treated with past or concurrent immunosuppressant therapy (including steroids) before the diagnosis of CS (data not shown), or those with high blood pressure.

**Table 3:** Strain Parameters on 2D–STE for Patients With Cardiac Sarcoidosis

Parameter	Value (*N* = 83)
LV GLS, %	−12.4 ± 3.7
LV GCS, %	−17.1 ± 6.5
LV GRS, %	29.3 ± 12.8
LV GLSR, s^−1^	−0.7 ± 0.2
LV GCSR, s^− 1^	− 1.1 ± 0.5
LV GRSR, s^−1^	1.7 ± 0.5
LV GLSRe, s^−1^	0.7 ± 0.2
LV GCSRe, s^−1^	1.2 ± 0.6
LV GRSRe, s^−1^	− 1.3 ± 1.0
RV GLS, %	−14.9 ± 6.3
RV GLSR, s^−1^	−0.9 ± 0.3
RV GLSRe, s^−1^	0.8 ± 0.3
RV free wall GLS, %	−16.2 ± 5.6
RV free wall GLSR, s^−1^	−1.1 ± 0.5
RV free wall GLSRe, s^−1^	0.95 ± 0.4

Abbreviations: *2D–STE* two-dimensional–speckle tracking echocardiography; *GCS* global circumferential strain; *GCSR* global circumferential strain rate; *GCSRe* early diastolic circumferential strain rate; *GLS* global longitudinal strain; *GLSR* global longitudinal strain rate; *GLSRe* early diastolic longitudinal strain rate; *GRS* global radial strain; *GRSR* global radial strain rate; *GRSRe* early diastolic global radial strain rate; *LV* left ventricular; *RV* right ventricular

### Comparison with patients without Sarcoidosis

Twenty-three patients with early stage of CS and LV systolic function within normal limits (LVEF> 52% for men; > 54% for women, mean value: 57.3 ± 3.8%) and no wall motion abnormalities or RV dysfunction at the time of CS diagnosis (mean age, 52.9 ± 10.0 years; 21.7% women; mean body mass index, 29.9 ± 4.7 kg/m^2^) were selected and their parameters compared with those of 97 controls (4:1 ratio) with normal echocardiographic findings and no history of coronary artery disease or other cardiovascular and metabolic comorbidities. At the time of cardiac evaluation, arterial blood pressure, linear dimensions of LV walls and LV mass were within normal limits. Only 16% of these CS patients had diastolic dysfunction (grade 1), and only 4 patients had mildly elevated LV filling pressures. No wall aneurysms or septal thinning were detected. Parameters of RV systolic function were normal in all patients (mean tricuspid S′, 0.13 ± 0.02 m/s and fractional area change 39.6% ± 9.4%). Mean pulmonary artery pressure was 52 ± 17 mmHg; no severe tricuspid or mitral valve impairment was detected, and only two CS patients had moderate tricuspid regurgitation. All 2D–STE and conventional systolic and diastolic echocardiographic parameters were significantly reduced when they were compared to those of controls (Table 4).

**Table 4:** Characteristics and Strain Parameters of CS Patients With Normal LV and RV Function and Control Patients Without CS^a^

Variable	CS Patients with LVEF > 52% (men) and > 54% (women) (*n* = 23)	Control Patients (*N* = 97)	*P* Value
Age, y	52.9 ± 10.0	40.4 ± 13.6	.0001
Female sex, No. (%)	5 (21.7)	61 (62.9)	.0001
BMI, kg/m^2^	29.9 ± 4.7	25.9 ± 4.8	.001
Systolic BP, mm Hg	123.3 ± 16.5	115.6 ± 15.5	.05
Diastolic BP, mm Hg	71.6 ± 7.2	71.2 ± 9.8	.81
Heart rate, bpm	75.3 ± 20.4	72.7 ± 13.3	.45
LV mass indexed for BSA, g/m^2^	92.7 ± 22.9	76.7 ± 13.6	.0001
LVEF, %	57.3 ± 3.8	63.9 ± 3.8	.0001
E/A	1.1 ± 0.6	1.6 ± 0.6	.001
E/e′	9.2 ± 4.4	7.7 ± 1.9	.01
LV GLS, %	−15.9 ± 2.5	−18.2 ± 2.7	.001
LV GCS, %	−22.5 ± 4.7	−21.9 ± 4.7	.60
LV GRS, %	37.1 ± 13.1	44.1 ± 12.1	.02
LV GLSR, s^−1^	−1.0 ± 1.2	−1.0 ± 0.2	.27
LV GCSR, s^−1^	−1.5 ± 0.4	− 1.4 ± 0.3	.22
LV GRSR, s^−1^	2.0 ± 0.4	2.2 ± 0.6	.09
LV GLSRe, s^−1^	0.9 ± 0.2	1.1 ± 0.2	.004
LV GCSRe, s^−1^	1.6 ± 0.6	1.4 ± 0.3	.08
LV GRSRe, s^−1^	−1.4 ± 1.3	−2.1 ± 0.8	.002
RV GLS, %	−16.9 ± 4.5	−24.1 ± 4.0	.0001
RV GLSR, s^−1^	− 1.1 ± 0.3	−1.4 ± 0.3	.0001
RV GLSRe, s^−1^	1.0 ± 0.3	1.5 ± 0.4	.0001
RV free wall GLS, %	−19.1 ± 5.1	−26.0 ± 5.2	.0001
RV free wall GLSR, s^−1^	− 1.3 ± 0.4	−1.6 ± 0.4	.003
RV free wall GLSRe, s^−1^	1.1 ± 0.4	1.9 ± 0.8	.0001

Abbreviations: *BMI* body mass index; *BP* blood pressure; *bpm* beats per minute; *BSA* body surface area; *CS* cardiac sarcoidosis; *E/A* ratio of early (E) to late (A) ventricular filling velocity; E/e′, ratio between early mitral inflow velocity and mitral annular early diastolic velocity; *GCS* global circumferential strain; *GCSR* global circumferential strain rate; *GCSRe* early diastolic circumferential strain rate; *GLS* global longitudinal strain; *GLSR* global longitudinal strain rate; *GLSRe* early diastolic longitudinal strain rate; *GRS* global radial strain; *GRSR* global radial strain rate; *GRSRe* early diastolic global radial strain rate; *LV* left ventricular; *LVEF* left ventricular ejection fraction; *RV* right ventricular

^a^ Mean ± SD unless otherwise indicated

### Diagnostic value of speckle tracking analysis

**Fig. 2:**
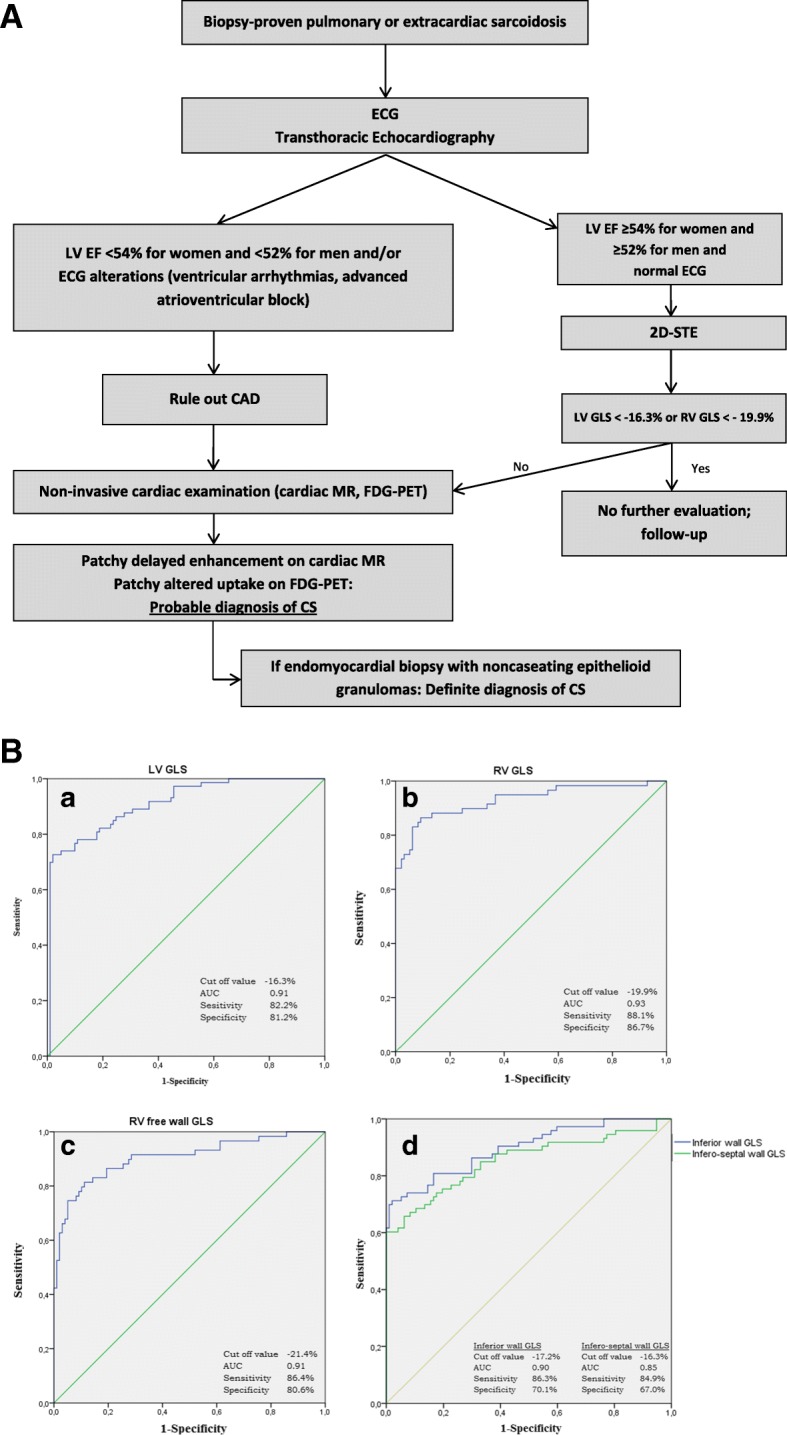
**a** Algorhythm for the diagnosis of cardiac sarcoidosis. **b** (Panel a,b,c,d). Receiver operating characteristic curves for global longitudinal strain (GLS) for patients (*n* = 83) affected by cardiac sarcoidosis. AUC: area under the curve; LV: left ventricle; RV: right ventricle; GLS: global longitudinal strain

**Fig. 3:**
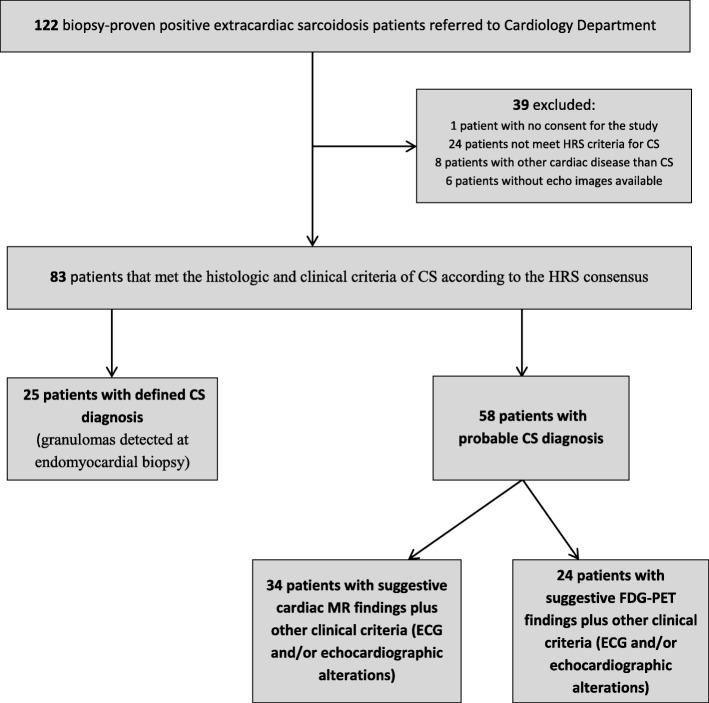
Design of the study. CS: cardiac sarcoidosis; HRS: Heart Rhythm Society; MR: magnetic resonance; FDG-PET, fluorodeoxyglucose-positron emission tomography

ROC analysis was conducted evaluating the diagnostic value of LV GLS and RV GLS for the identification of cardiac involvement of sarcoidosis. As shown in Fig. 2a&b, a LV GLS value of − 16.3% provided 82.2% sensitivity and 81.2% specificity for the diagnosis of CS (area under the curve, [AUC] 0.91); An inferoseptal GLS value of − 16.3% provided 84.9% sensitivity and 67% specificity for the diagnosis of CS (AUC 0.85); when the inferior wall was added to the model, the sensitivity increased to 86.3% and specificity to 70.1% (AUC: 0.90). A RV GLS value of − 19.9% provided 88.1% sensitivity and 86.7% specificity (AUC 0.93), while a free wall RV GLS of − 21.4% provided 86.4% sensitivity and 80.6% specificity (AUC 0.91) Fig. 3. LVEF, LV GCS, and LV GRS did not offer additional diagnostic value for CS.

Intra-class correlation coefficients were the highest for global longitudinal strain measurements, followed closely by circumferential strain measurements, and the lowest for global radial strain measurements. GLS 0.998 (0.994–0.999), 0.96 (0.989–0.998), GCS 0.997 (0.994–0.999), 0.993 (0.983–0.997) and GRS 0.857 (0.751–0.933) and 0.845 (0.727–0.927) for intra-observer and inter-observer respectively.

### Outcomes

Median follow-up was 26.3 months (IQ25% 9.59, Q75% 68.2 months, range 1–226.2). An LV GLS value more positive than − 14% was found to be related to a higher rate of hospital admission for cardiac complications (OR, 4.17 [95% CI, 1.34–12.98]; *P* = .01) and heart failure (OR, 5.03 [95% CI, 1.04–21.32]; *P* = .04) in the univariate logistic regression analysis. Reduced LVEF or abnormal ECG findings did not correlate with cardiac events (data not shown).

## Discussion

To our knowledge, this is the first study to describe cardiac mechanics assessed by 2D–STE in patients with a diagnosis of CS according to HRS criteria. In our cohort, reduced LV and RV regional myocardial deformation parameters were detected for almost all CS patients, regardless of the procedure used for diagnosis (histology, clinical examination, heart rhythm criteria, MR or FDG-PET imaging)—even in those patients with normal conventional parameters of LV and RV systolic function. Strain parameters were worse for patients with more advanced stages of CS, in particular for those patients with severe systolic dysfunction diagnosed by endomyocardial biopsy. In patients with impaired LV GLS (> − 14%), hospital admission and heart failure were increased significantly. Nevertheless, in our study, strain parameters were not significantly influenced by treatment with immunosuppressant medications. These findings are consistent with those of previous studies that evaluated data for asymptomatic patients with extracardiac sarcoidosis and no definite cardiac involvement [20–23]. However, in these reports, alterations in regional myocardial mechanics attributable to other noncardiac causes (ie, pulmonary hypertension, valve disease, or concurrent steroid treatment) could not be excluded. In addition, 2D–STE was able to detect indirect signs of myocardial damage (both active inflammation or scars and fibrosis) seen on CMR or FDG–PET imaging [24, 25]. More studies are needed to confirm our findings.

The best diagnostic assessment of CS is still being debated. Suggested annual routine screening that includes a physical examination and history and 12-lead ECG has been suggested, but this approach has several limitations [26]. In our cohort, most CS patients reported cardiac symptoms, in particular palpitations and dyspnea. However, these symptoms are often nonspecific and lack predictive value in diagnosing CS [27]. In addition, abnormal basal ECG findings were found to have low predictive value for diagnosing CS [10, 28]. Among radiologic imaging techniques, CMR can identify early myocardial alterations and distinguish among inflammatory, edematous, and fibrotic phases with high sensitivity and good accuracy [29, 30]. FDG–PET has also shown good accuracy for diagnosing CS [31]. However, these types of imaging studies as screening tests have high cost. As shown in our study, CS is often diagnosed months or years after extracardiac sarcoidosis is detected, and many patients undergo implantable defibrillator or pacemaker implantation after they experience life-threatening ventricular arrhythmias or have advanced atrial-ventricular blocks.

Regarding transthoracic echocardiography, conventional parameters of ventricular systolic and diastolic function have suboptimal sensitivity and specificity, especially in early phases of CS [7]. We detected interventricular septal thinning in 13.3% and wall aneurysms in 4.9% of CS patients, 2 of the typical echocardiographic findings seen in advanced stages of the disease [32]. Furthermore, we did not identify any interventricular septal thickening, a rare presentation described previously [33]. However, in our cohort, 2D–STE could detect early systolic dysfunction and alterations in regional myocardial deformation, even in patients with normal conventional parameters of systolic function at an early stage of the disease [34]. Our study showed high sensitivity of LV GLS, as well as LV inferior, inferoseptal wall GLS, and RV and RV free wall GLS for CS diagnosis, suggesting that these values should be considered in the evaluation of CS. Reduced GLS should be followed up with further noninvasive radiologic imaging. Abnormal strain parameters may be useful in detecting isolated CS, especially in patients who do not meet current HRS diagnostic criteria [35].

In our CS patients, we detected a high rate of cardiac adverse events. This finding is similar to others reported in the literature [36, 37]. In our cohort, reduced LV GLS predicted hospital admission and heart failure; furthermore, CS patients with normal strain values did not have adverse outcomes such as death or stroke. They also did not require a heart transplant or left ventricular assist device. Thus, CS patients with lower GLS values should be closely monitored because of a higher risk of adverse cardiac events. In addition, early diagnosis of myocardial involvement with 2D–STE may also be helpful for identifying early phases of the disease characterized by myocardial inflammation and edema. When CS is identified early, patients could be treated earlier and probably the progression of CS and development of severe end-stage cardiomyopathy or arrhythmic events may be prevented.

### Limitations

This is a single-center retrospective study. Further prospective studies are needed to validate the value of 2D–STE for CS. Most patients referred to our clinic had suspected cardiac involvement because of symptoms or nonspecific ECG findings, so we could have overestimated the prevalence of CS in early stages. Otherwise, we cannot exclude a referral bias, because most of the CS patients enrolled were followed in our Pulmonary Medicine Clinic for pulmonary complications.

Unfortunately, not all patients underwent simultaneous advanced imaging (both FDG-PET and CMR) and concurrent transthoracic echocardiography. No CMR or FDG-PET data are available for the healthy control group. We had no data on pulmonary function tests or the degree of pulmonary involvement from sarcoidosis; however, Mehta et al. [10] did not find any correlation between degree of pulmonary involvement and the presence of CS. In addition, the short-term follow-up may have underestimated the prevalence of adverse events.

## Conclusions

In patients with CS, Abnormal LV GLS, inferoseptal and inferior wall strain and RV GLS are good predictors of CS even if LVEF or RV systolic parameters are within normal limits. Furthermore, reduced LV strain parameters (GLS more positive than − 14%) correlated with heart failure and hospital admission. 2D–STE is a sensitive method for the early detection of subclinical myocardial involvement of sarcoidosis, particularly when CMR and FDG–PET imaging are contraindicated or not readily available.

## Data Availability

The datasets generated and/or analyzed during the current study are not publicly available because the information and data of the study population were extracted from Hospital Information System and were recorded manually in EXCEL to form our private database. But the data are available from the corresponding author on reasonable request.

## Abbreviations

2D: Two-dimensional
2D–STE: Two-dimensional−speckle tracking echocardiography
A: Peak velocity of late transmitral flow
AUC: Area under the curve
AV: Atrioventricular
CMR: Cardiac magnetic resonance
CS: Cardiac sarcoidosis
E: Peak velocity of early diastolic transmitral flow
ECG: Electrocardiogram e′ velocities, mitral annular early diastolic velocity
FDG–PET: Fluorodeoxyglucose–positron emission tomography
GCS: Global circumferential strain
GCSRe: Global circumferential early diastolic strain rate
GCSRs: Global circumferential systolic strain rate
GLS: Global longitudinal strain
GLSRe: Global longitudinal early diastolic strain rate
GLSRs: Global longitudinal systolic strain rate
GRS: Global radial strain
HRS: Heart Rhythm Society
LV: Left ventricle
LVEF: Left ventricular ejection fraction
OR: Odds ratio
ROC: Receiver operating characteristic
RV: Right ventricle
RWT: Relative wall thickness
S′: Peak systolic annular velocity and HL
SR: Strain rate
SRe: Early diastolic strain rate
SRs: Systolic strain rate
